# Supplementary material for: Three-Dimensionally Printed Expandable Structural Electronics Via Multi-Material Printing Room-Temperature-Vulcanizing (RTV) Silicone/Silver Flake Composite and RTV
Source: Polymers (Basel). 2023 Apr 23;15(9):2003. doi: 10.3390/polym15092003 (PMC10181061; doi:10.3390/polym15092003)

Figure S1. Particle size distribution of Ag flakes. Particle size distribution displayed in volume weighted version.

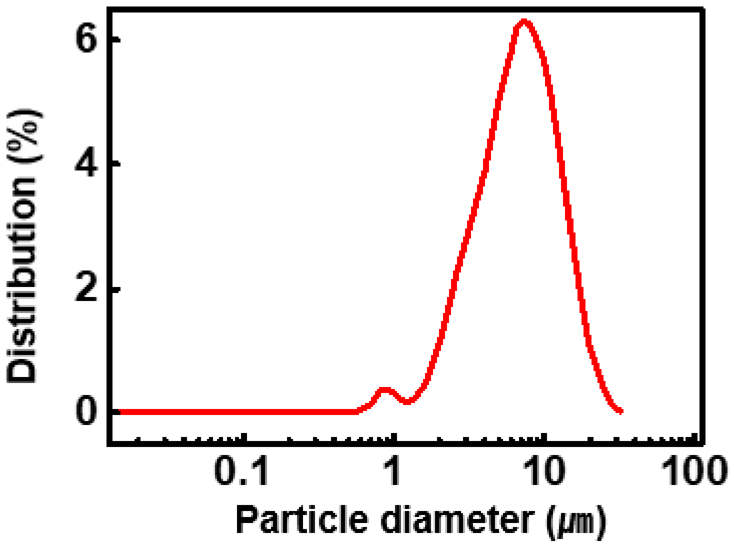

Figure S2. Adhesion test of sRTV to various substrates. Substrates are glass, Cu tape, polyimide tape, polypropylene tape, release agent treated glass from left to right. Diameter of 1 cm circle is printed on each substrate (a) Right after printing (b) Left surface after peel-off

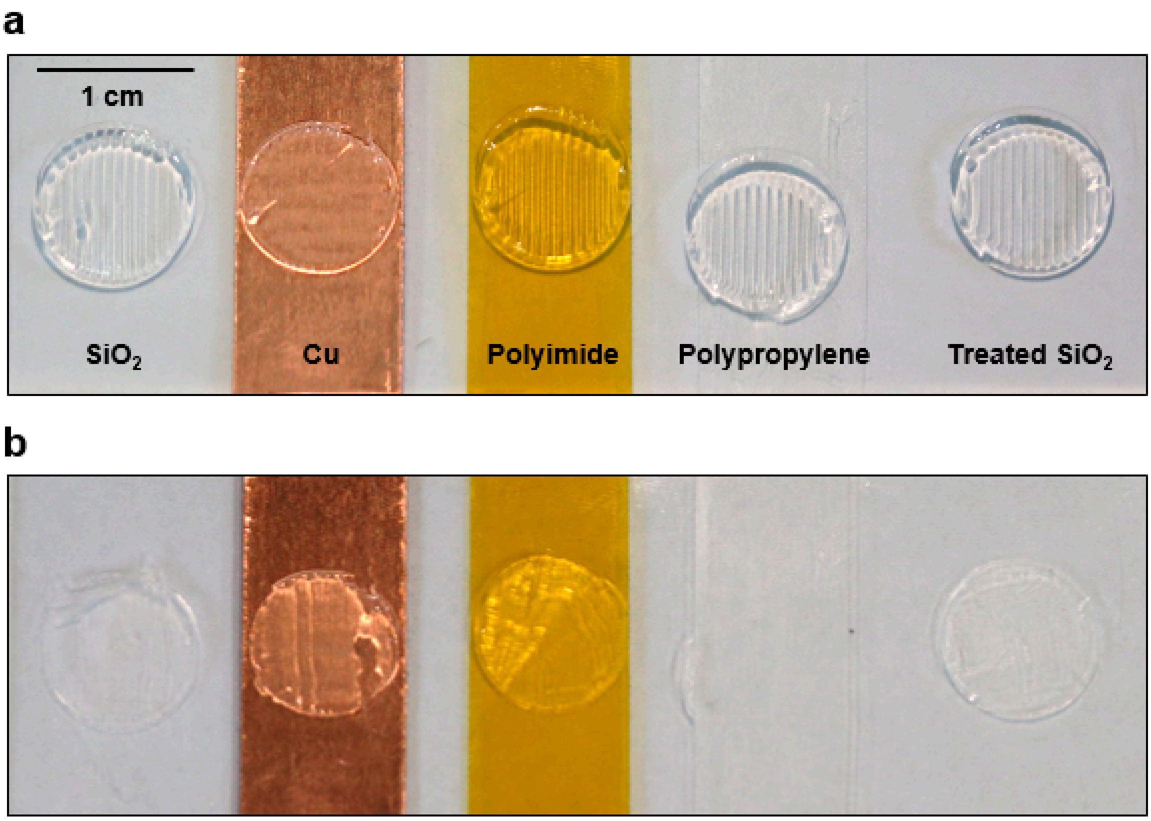

Figure S3. Various 3D structure printed with sRTV as frame materials. (a) Triangular horizontal 1 channel (b) Hexagonal vertical 3 channel

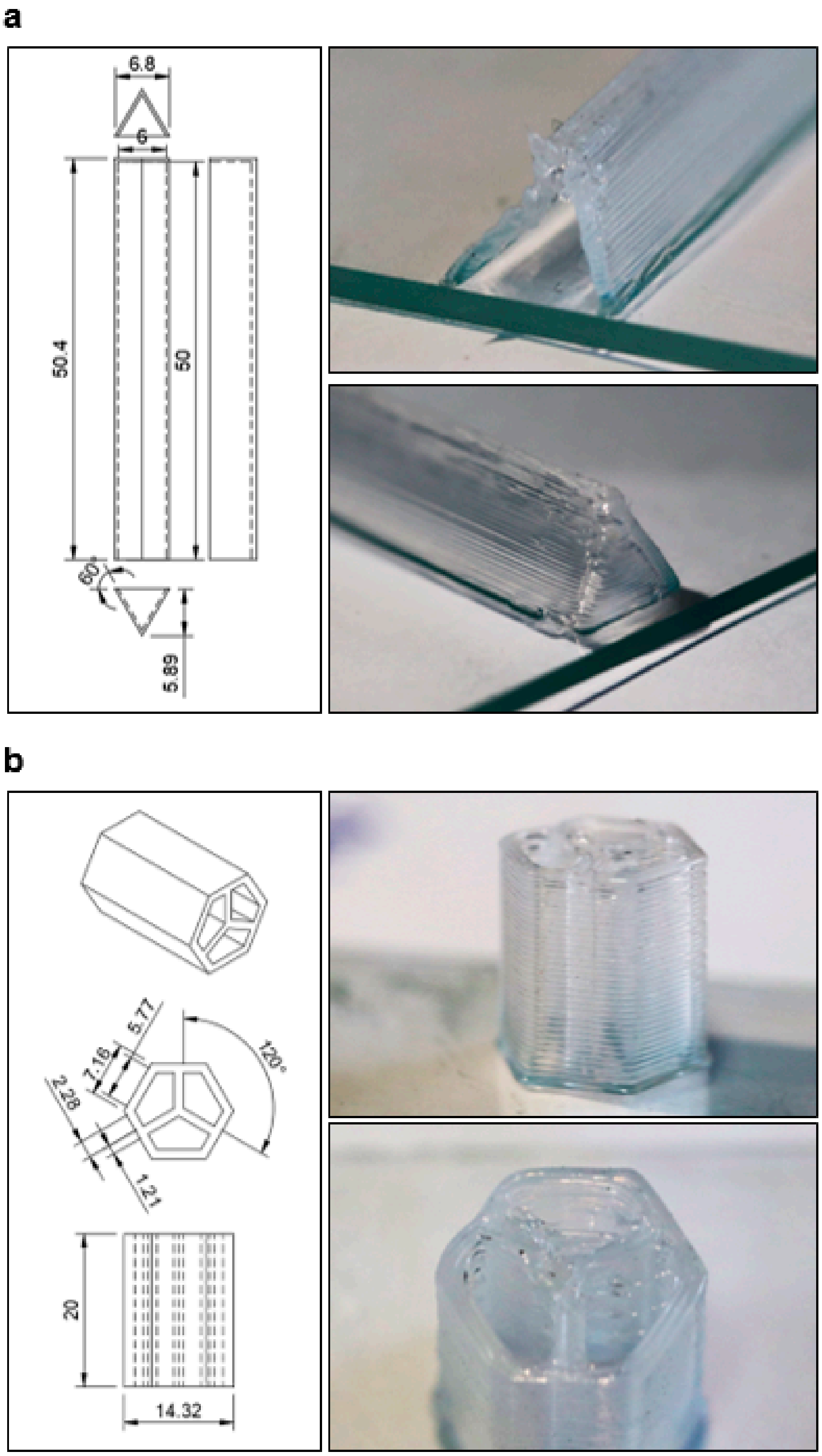

Figure S4. Rheological properties and 3D printability of rRTV. (a) Shear modulus change by shear stress (b) Viscosity change by shear rate (c) 1 cm diameter and 5 mm height scaffolds with grid pattern with rRTV

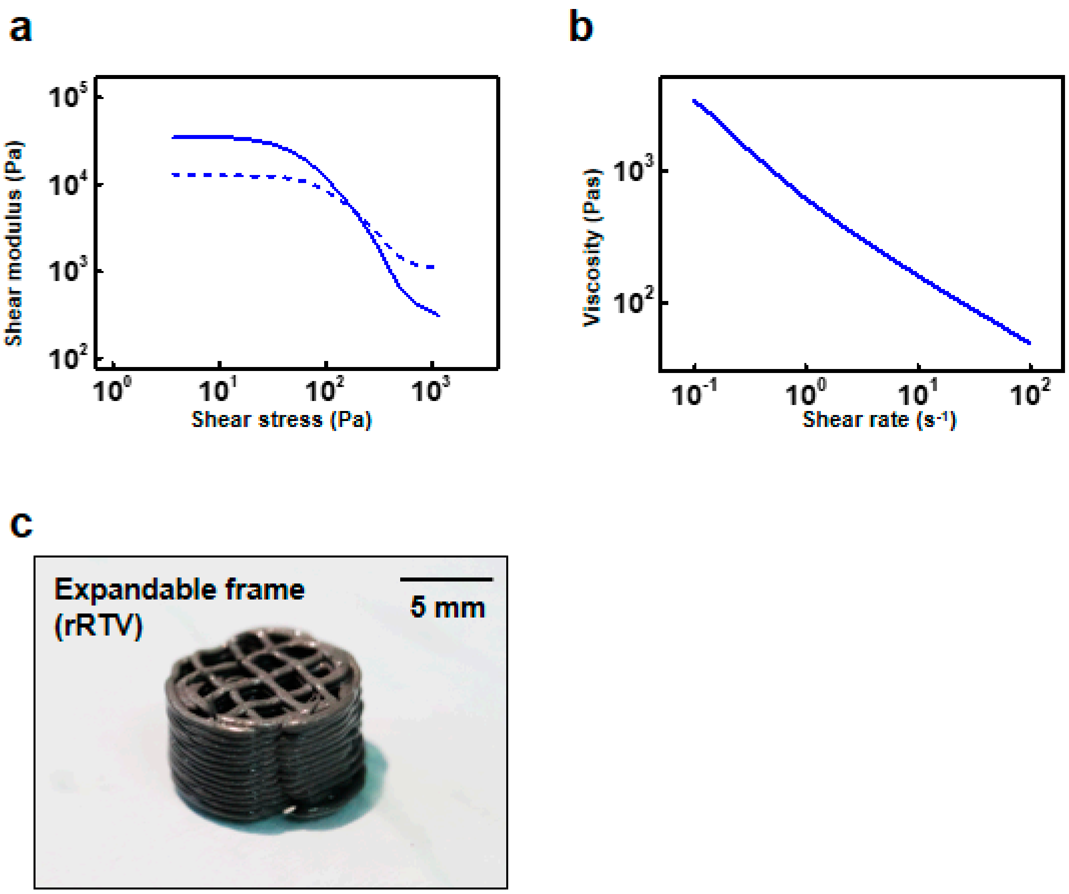

Figure S5. Stress-Strain curve of rRTV.

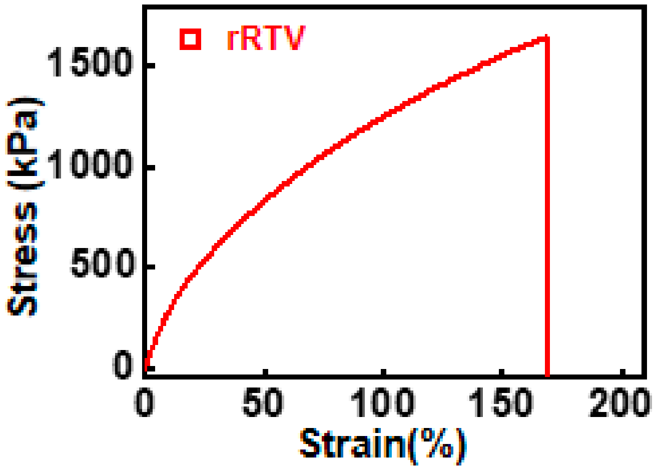

Figure S6. Resistance change of Ag-RTV in various environments. (Black, HCL 20 % ; Red, H_2SO_4 20 %; Blue, NaOH 20 %; Margenta, PBS 1X)

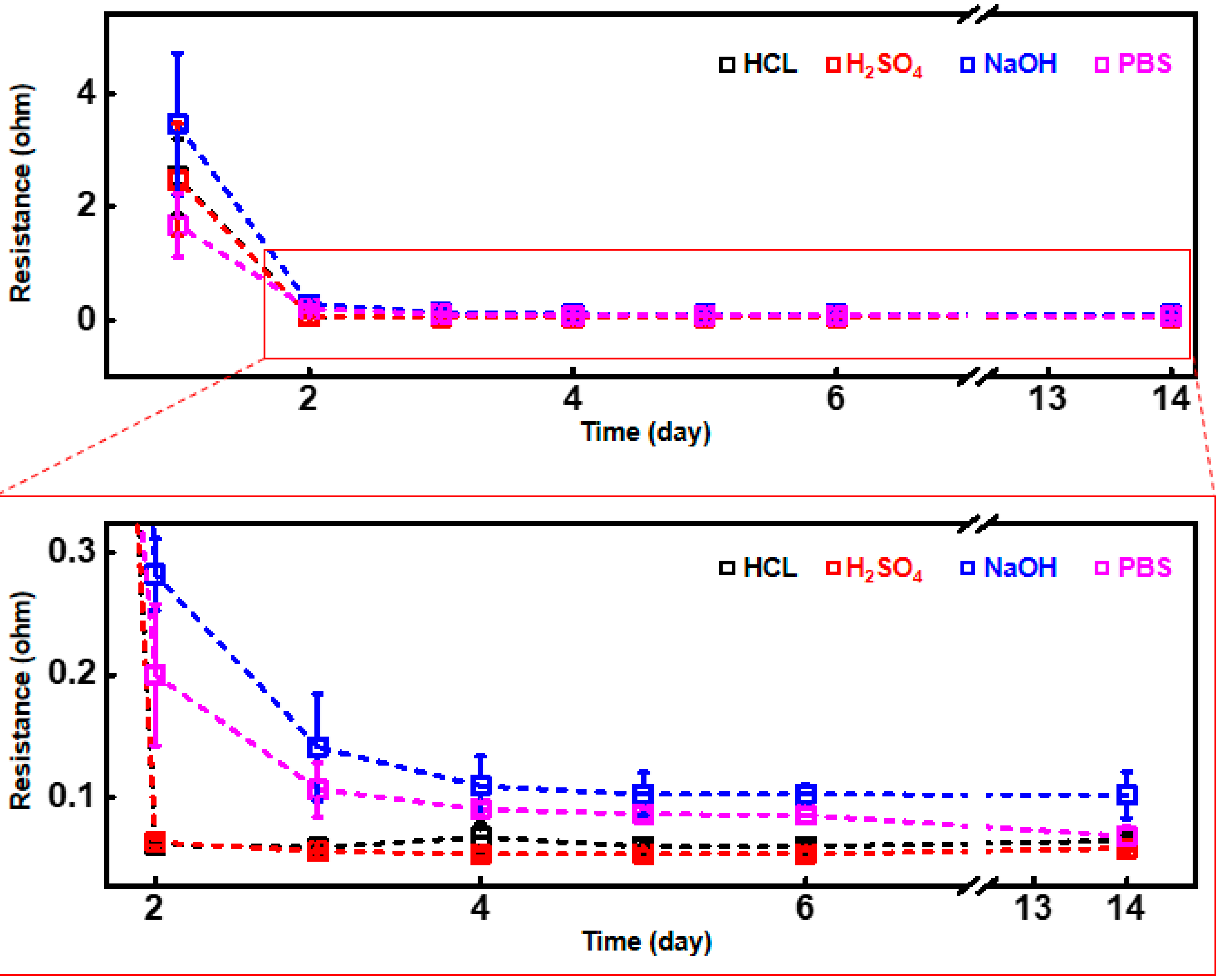

Figure S7. Dimension of auxetic structured substrate. (a) Unit cell of missing rib structures where r = 2.121 mm, width of line = 0.4 mm (b) Three types of auxetic pattern for tensile test sample where height is 29 mm and width is 30 mm.

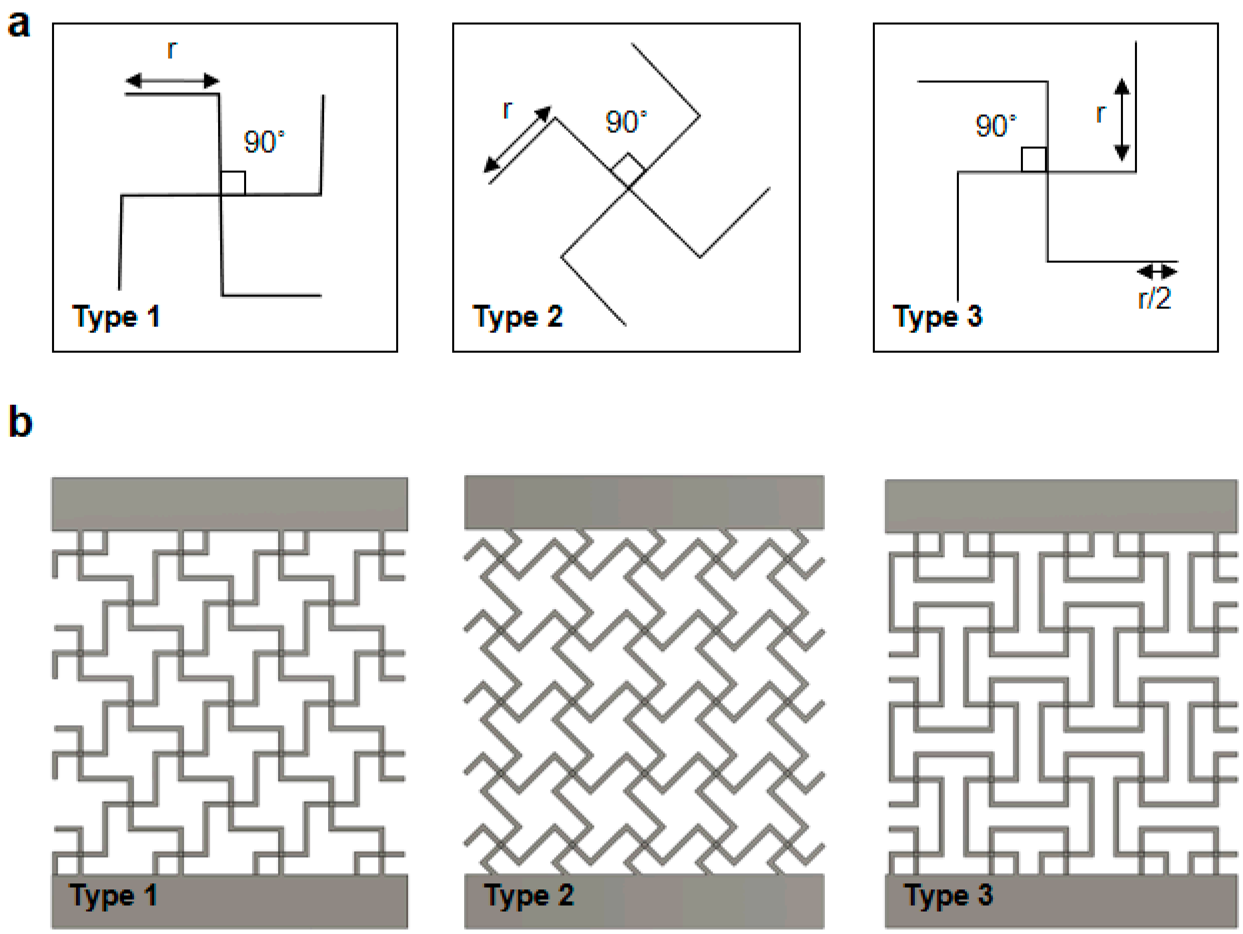

Figure S8. SEM images of Ag-RTV trace morphology before and after uniaxial tensile test. (a) top view and (b) cross section view of Ag-RTV trace before stress applied and (c) top view and (d) cross section view of Ag-RTV trace after mechanical failure.

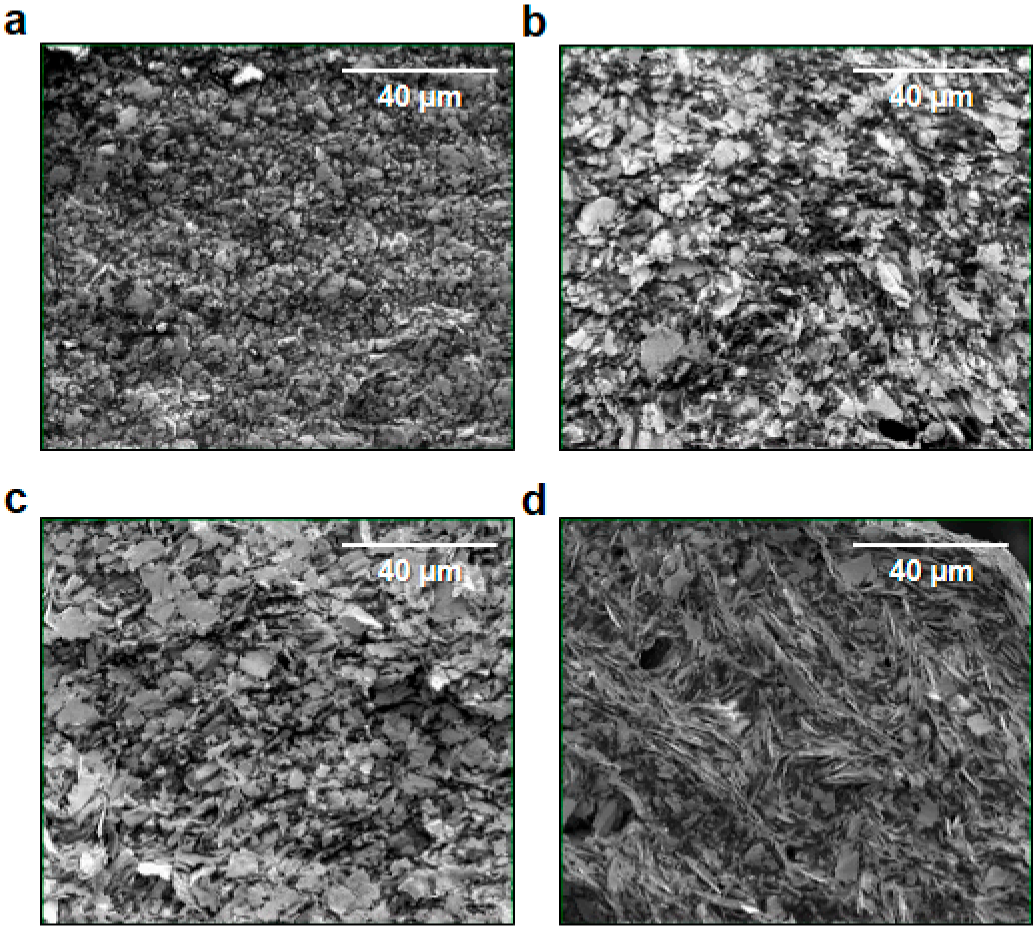

Figure S9. Poisson's ratio calculation with sRTV and rRTV. (a) Serial images of type 1 missing rib arrays with sRTV and rRTV in strain 0 %, 10 %, 20 % (b) Strain dependent Poisson's ratio with sRTV and rRTV (c) Box chart of Poisson's ratio ranges for sRTV and rRTV for strain 2.5 % to 20 %. Box ranges from 25 to 75 %, - for max / min, × for 99 % / 1 %, □ for mean value.

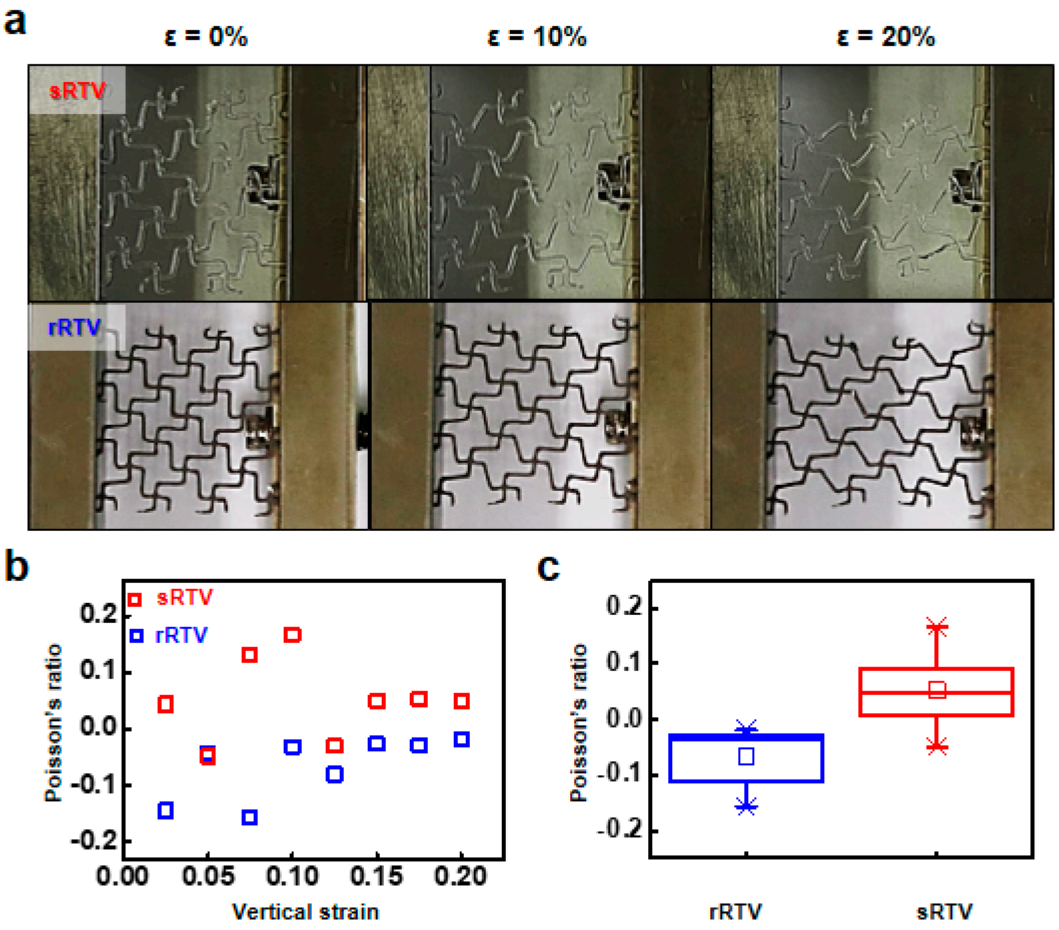

Figure S10. G-codes for capacitive strain sensor in missing rib structure with Ag-RTV, rRTV. (a) Capacitor electrode 1 (b) Capacitor electrode 2 (c) Control frame

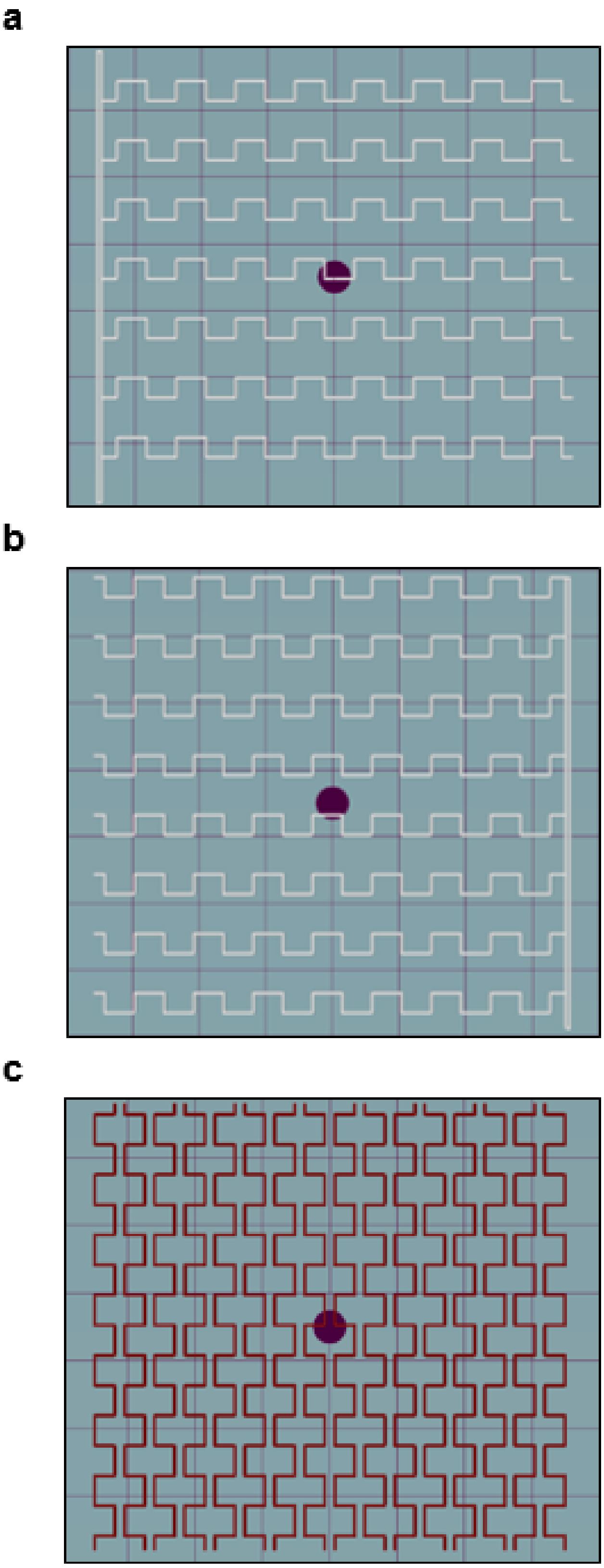

Figure S11. G-codes for resistive heater in missing rib structure with Ag-RTV and rRTV. (a) Resistor (b) Control frame

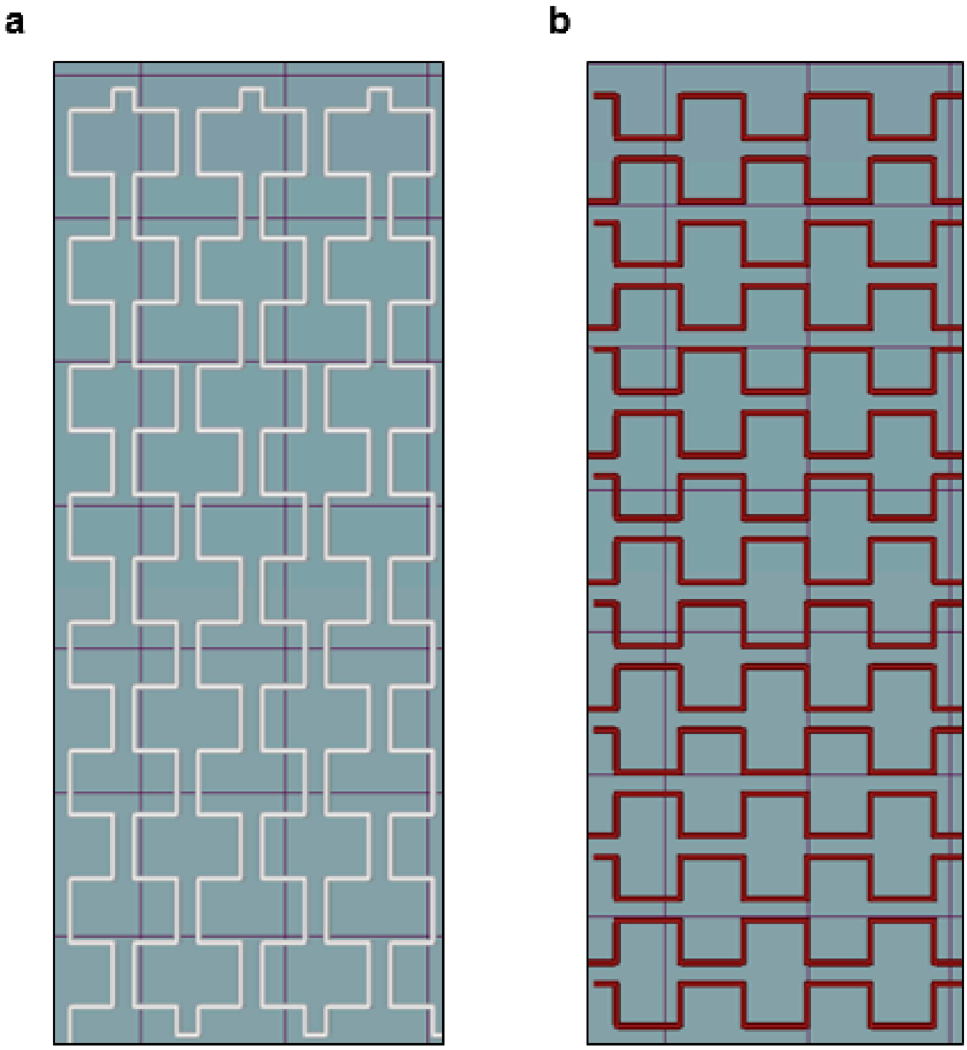

Figure S12. Setup for electromechanical properties measurements.

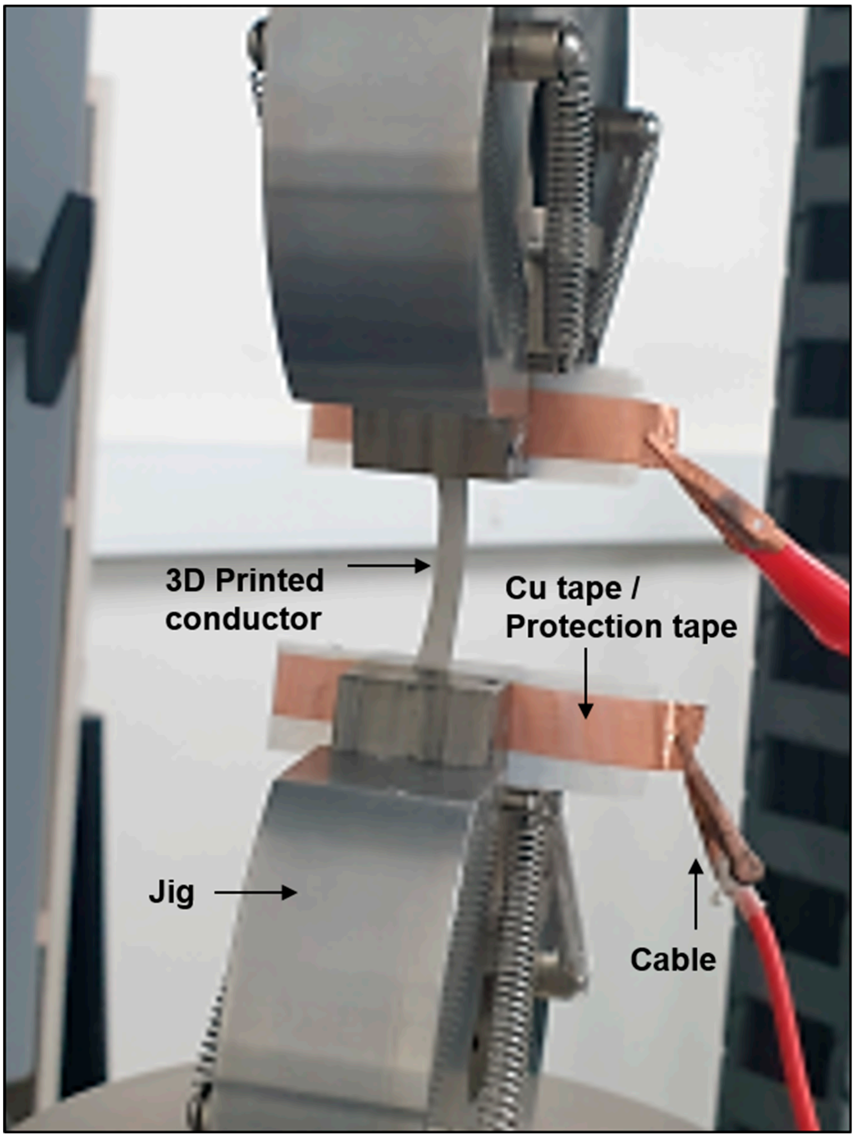

Figure S13. Expandable structure effects on resistance of Ag-RTV in strain change 0 % to 25 %.

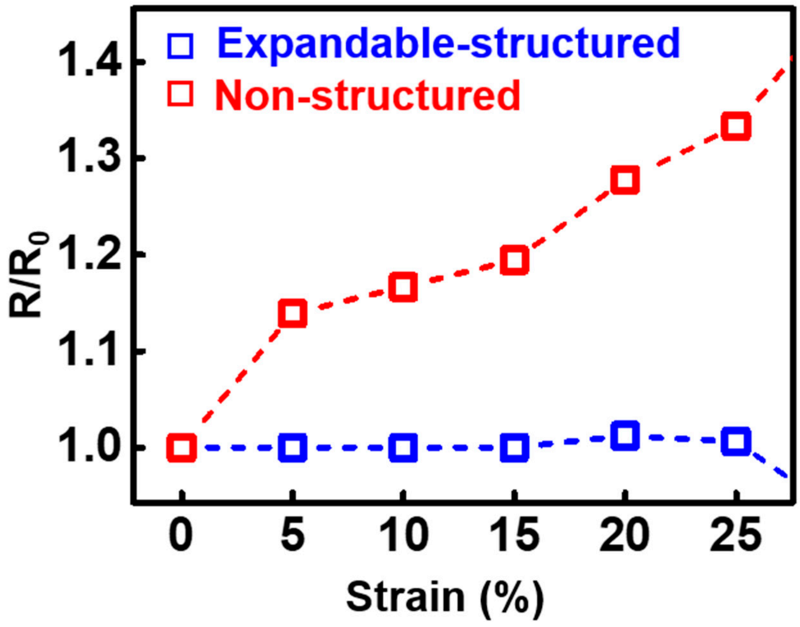

Figure S14. 3D printed stretchable inductor (a) Image of printed inductor (Ag-RTV/sRTV) (b) Inductance and quality factor of 3D printed inductor by frequency.

a

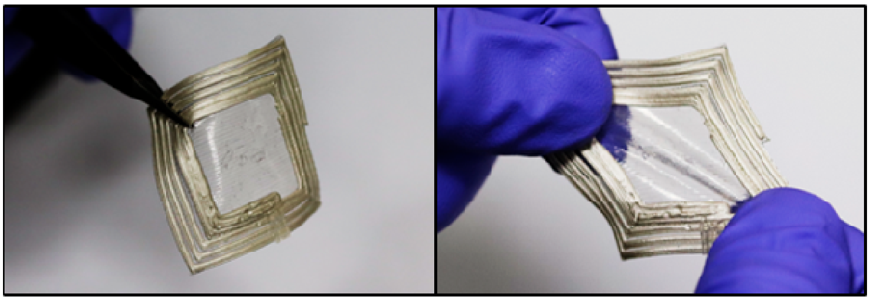

b

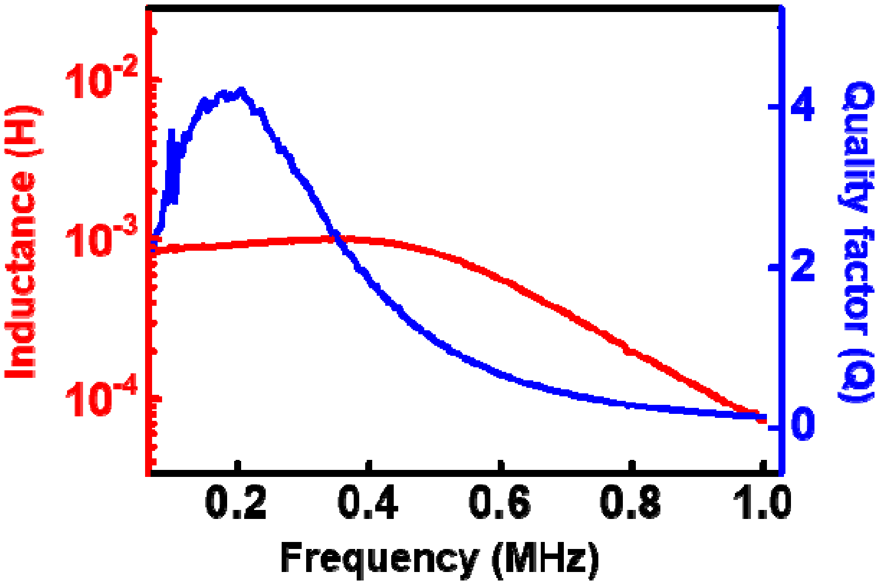

Supplement: Supplementary file 1 [file polymers-15-02003-s001.zip › polymers-2364869-supplementary.pdf]
